# Glycerine as a Therapeutic Agent

**Published:** 1876-09

**Authors:** 


GLYCERINE AS A THERAPEUTIC AGENT.—Dr. C. H. Lidd states that children raised by hand can be kept comparatively free from many of the ills to which they are subject, if from four to six drachms daily of glycerine be substituted for sugar. The exosmotic property of glycerine surpasses those of any agent at our command, and has the property of withdrawing the watery elements from any tissue to which it is continuously applied. Marion Sims showed that a ball of lint saturated with glycerine will arrest the secretion of a suppurating surface; and Furst and Eberhurd state they have successfully used a glycerine plug in fluor albus.—*Edinburg Med. Journ.*

